# PD-L1 expression in high-risk non-muscle invasive bladder cancer is not a biomarker of response to BCG

**DOI:** 10.1007/s00345-024-05392-5

**Published:** 2025-01-03

**Authors:** Florus C. de Jong, Vebjørn Kvikstad, Robert F. Hoedemaeker, Angelique C. J. van der Made, Thierry P. van der Bosch, Niels J. van Casteren, Kim E. M. van Kessel, Ellen C. Zwarthoff, Joost L. Boormans, Tahlita C. M. Zuiverloon

**Affiliations:** 1 Department of Urology, Erasmus University Medical Center, Erasmus MC Cancer Institute, Dr. Molewaterplein 40, Room Be-304, 3015 GD Rotterdam, The Netherlands; 2 Department of Pathology, Helse Stavanger HF, Stavanger, Norway; 3 Pathan BV, Department of Pathology, Rotterdam, The Netherlands; 4 Department of Pathology, Erasmus University Medical Center, Erasmus MC Cancer Institute, Rotterdam, The Netherlands; 5 Department of Urology, Franciscus Gasthuis & Vlietland, Rotterdam, The Netherlands; 6 Department of Urology, Amphia, Breda, The Netherlands

**Keywords:** BCG, Bladder cancer, Immunotherapy, PD-L1, Prognosis, Progression, Recurrence

## Abstract

**Purpose:**

Up to 50% of high-risk non-muscle invasive bladder cancer (HR-NMIBC) patients fail Bacillus Calmette-Guérin (BCG) treatment, resulting in a high risk of progression and poor clinical outcomes. Biomarkers that predict outcomes after BCG are lacking. The antitumor effects of BCG are driven by a cytotoxic T cell response, which may be controlled by immune checkpoint proteins like Programmed Death Ligand 1 (PD-L1). Here, we hypothesized that PD-L1 protein expression could serve as a biomarker for BCG-failure.

**Methods:**

HR-NMIBC patients who received ≥ 5 BCG instillations were included. Tissue microarrays were constructed from BCG-naïve tumors and recurrences and stained with the PD-L1 (SP142) antibody. PD-L1 status was defined as ≥ 5% tumor-infiltrating immune cells with membrane staining in the tumor area. Clinicopathological associations with PD-L1 positive tumors were investigated, and time-to-event analyses were performed comparing PD-L1 positive vs. negative tumors.

**Results:**

432 BCG-naïve tumors and 160 recurrences were included, and 91% of patients received adequate BCG. In BCG-naïve tumors, PD-L1 was expressed in 7% of patients and PD-L1 expression was associated with stage T1 versus Ta disease (*p* = 0.015). PD-L1 expression was not associated with treatment failure after adequate BCG (*p* = 0.782) nor with progression-free survival (*p* = 0.732). Testing cut-offs of ≥ 1% and ≥ 10% PD-L1 positivity did not alter results. High PD-L1 expression was more frequent in tumor recurrences (14%) as compared to BCG-naïve tumors (*p* = 0.012).

**Conclusion:**

PD-L1 expression in HR-NMIBC is not a biomarker of response to BCG. However, PD-L1 is higher in a subset of tumors that failed BCG treatment. More research is needed to determine the role of PD-L1 in tumors where BCG treatment failed.

**Supplementary Information:**

The online version contains supplementary material available at 10.1007/s00345-024-05392-5.

## Introduction

Current bladder cancer (BC) risk stratification identifies patients at risk of recurring and progressive disease. The standard-of-care for patients with high-risk non-muscle invasive BC (HR-NMIBC) includes a transurethral resection of the bladder tumor (TURBT) followed by adjuvant intravesical instillations with Bacillus Calmette-Guérin (BCG) for up to three years [1]. Despite treatment, up to 50% of patients experience, recurrences, and 10–20% develop progression, which is associated with poor clinical outcomes [2]. No markers exist to predict response to treatment. Patients who do not respond to BCG suffer from BCG toxicity and a delay in surgery—a radical cystectomy (RC) with urinary diversion [1]. An early RC instead of BCG is associated with excellent long-term outcomes [3]. However, early RC in all patients results in overtreatment. Furthermore, most patients prefer bladder-sparing therapy since RC is associated with a reduced quality of life [4].

The cell surface protein Programmed Death Ligand 1 (PD-L1) binds to the Programmed Death 1 (PD-1) receptor on CD8 + T cells and suppresses a Th-1 immune response by inducing CD8 + T cell apoptosis, thereby suppressing antitumor immunity [5]. The antitumor effect of BCG is effectuated via a cytotoxic T cell response against residual tumor cells after TURBT [6]. Hence, it is hypothesized that the diminished effectivity of BCG therapy may be caused by upregulated PD-L1 expression, which could serve as a marker for lack of response to BCG. Evidence suggests that PD-L1 expression in tumor cells is associated with BC stage progression and poor clinical outcomes in advanced BC, but this is not the case for HR-NMIBC [7–10]. A recent review highlighted conflicting evidence and a lack of detailed information on BCG treatment [11]. As a result, the role of PD-L1 as a biomarker for predicting response to BCG in NMIBC is not fully established.

The role of PD-(L)1 immune checkpoint inhibitors (ICI) is also significant in BCG-unresponsive patients. *Pembrolizumab* was approved as monotherapy based on results from the phase 2 Keynote-057 study, but only 41% of patients had a complete response at 3 months [12]. Improving the success rate for ICIs by identifying patients who would benefit from ICI remains an unmet clinical need [13].

Here, we aimed to investigate if immunohistochemical (IHC) expression of PD-L1 can predict response to BCG in a large cohort of BCG-treated patients with pathological revision and long-term follow-up. In addition, we discuss if PD-L1 expression could provide a rationale for anti-PD-(L)1 ICI therapy in BCG-naïve tumors and recurrences.

## Patients and methods

### Patients

Patients with primary HR-NMIBC (Ta high-grade [HG]/T1/T*is*) who received ≥ 5/6 BCG induction instillations were retrospectively included in the study between 2000 and 2018. Recruitment occured at four hospitals: Erasmus University MC, Franciscus Gasthuis & Vlietland and Amphia (all in the Netherlands), and Stavanger University Hospital (in Norway). All urologists were recommended to follow the then applicable European Association of Urology (EAU) guidelines on NMIBC during this study period; detailed follow-up protocols involving cystoscopy, cytology, and imaging (e.g. for upper tract and/or progressive disease) can be found in the supplemental methods. The BCG instillation schedule followed the Southwest Oncology Group (SWOG) protocol [1]. Follow-up concluded at the last visit or death, and was completed in September 2021.

### Pathology

A large cohort (*n* = 509) of centrally reviewed HR-NMIBC tumors, including tumor recurrences, was used to construct tumor tissue microarrays (TMAs). Preferred regions of interest (ROI) were invasive tumor areas with tumor-infiltrating immune cells (ICs). The anti-PD-L1 antibody (VENTANA PD-L1 (SP142) Assay, Ventana Medical Systems, the companion diagnostic for *atezolizumab*) was used for IHC of TMAs. PD-L1/SP142 IHC was evaluated by two independent investigators, who were blinded to clinical outcomes during the assessment. Details on TMA construction, assessment, and IHC are provided in the supplements.

### Definitions, endpoints & statistics

Adequate BCG was defined as receiving ≥ 5/6 BCG induction instillations plus ≥ 2/3 BCG maintenance instillations [14]. BCG failure was determined according to the 2021 EAU Guidelines on NMIBC [1]. The primary endpoint was defined as: a HG recurrence after the second round of BCG instillations (~ 6 months after induction), accounting for patients with a TaHG/Tis recurrence who may undergo a second round of BCG instillations. Patients who developed T1HG disease after BCG induction were also included in the primary endpoint. Secondary endpoints included recurrence at the first evaluation after BCG induction, 1-year high-grade recurrence-free survival (HG-RFS), 2-year progression-free survival (PFS), and 5-year cancer-specific survival (CSS). Progression was defined as the development of MIBC and/or (lymph) node metastatic disease. Chi-square testing was done to assess if PD-L1 expression was associated with clinicopathological parameters, and to determine if PD-L1 status differed between tumor recurrences vs BCG-naïve tumors or post-BCG random biopsies (in case of suspected recurrences) diagnosed as pT0. The Kaplan–Meier method with log-rank testing was used to estimate survival. The time variable was the duration between initial cancer diagnosis and HG recurrence, progression, or death from BC. Outcomes were deemed significant at *p* < 0.05. Analyses were done in *R* statistical software (v4.0.5) and SPSS Statistics (IBM^®^ v26).

## Results

### Baseline study population and risk factors of BCG treatment failure

In BCG-naïve patients, N = 432 tumors with 1060 tumor cores were of sufficient quality for PD-L1 IHC, averaging approximately 2.5 cores per primary tumor. In 24/432 (%) tumors, PD-L1 information was used from the re-TURBT. Clinicopathological characteristics for included patients are summarized in Table 1. Ninety-one percent received adequate BCG, with a median clinical follow-up of 70 (IQR 45–98) months. According to the EAU guidelines on NMIBC, BCG treatment failed in 135/432 (31%) patients. Categories of BCG treatment failure are specified in Table 1. At baseline, tumor focality (*p* = 0.001), CIS (*p* = 0.03), LVI (*p* = 0.001), and the EAU very high-risk disease category (*p* = 0.003) were significantly correlated with BCG treatment failure.

**Table 1:**
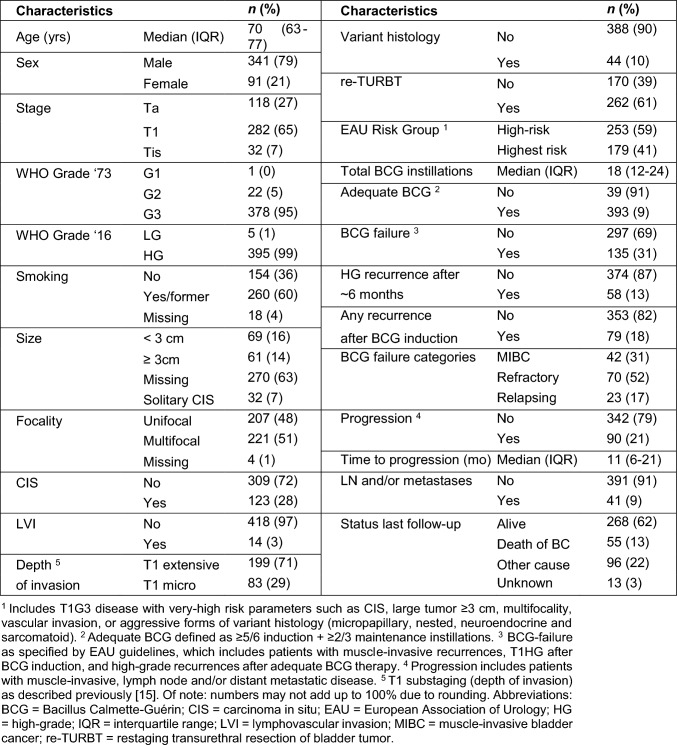
Baseline patient and follow-up characteristics of N = 432 BCG-naïve high-risk non-muscle invasive bladder cancer patients

*BCG* Bacillus Calmette-Guérin, *CIS* carcinoma in situ, *EAU* European Association of Urology, *HG* high-grade, *IQR* interquartile range, *LVI* lymphovascular invasion, *MIBC* muscle-invasive bladder cancer, *re-TURBT* restaging transurethral resection of bladder tumor

^a^Includes T1G3 disease with very-high risk parameters such as CIS, large tumor ≥ 3 cm, multifocality, vascular invasion, or aggressive forms of variant histology (micropapillary, nested, neuroendocrine and sarcomatoid)

^b^Adequate BCG defined as ≥ 5/6 induction + ≥ 2/3 maintenance instillations

^c^BCG-failure as specified by EAU guidelines, which includes patients with muscle-invasive recurrences, T1HG after BCG induction, and high-grade recurrences after adequate BCG therapy

^d^Progression includes patients with muscle-invasive, lymph node and/or distant metastatic disease

^e^T1 substaging (depth of invasion) as described previously [15]. Of note: numbers may not add up to 100% due to rounding

### BCG-naïve HR-NMIBC patients have a low protein expression of PD-L1

We analyzed the number of cores with PD-L1 staining in any type of tumor-infiltrating IC. An example of a negative core and a positive core with PD-L1 staining in ≥ 5% ICs is illustrated in Figure S1. Using the recommended cut-off of ≥ 5% in ICs, we found that 29/432 (7%) of BCG-naïve tumors were considered PD-L1 positive based on the mean value of all cores. Due to known PD-L1 staining heterogeneity, we also investigated which fraction of cores scored positive if the core with the highest value was selected to determine PD-L1 status. Using the highest core, 69/432 (16%) tumors were positive for PD-L1 expression. The SP142 antibody is designed for ICs, but as suggested by the protocol and others, we also investigated staining on tumor cells (TCs). Using a ≥ 5% cut-off, 19/432 (4%, mean core expression) vs 40/432 (9%, highest expressed core) tumors were considered positive. All results on PD-L1 expression in ICs and TCs and other cut-offs used in literature are included in Table S1. To ensure that our findings were not influenced by PD-L1 spatial heterogeneity and the use of TMAs, we selected 30 samples (15 T1 responders vs. 15 non-responders to BCG) and checked if PD-L1 status based on TMAs differed from whole slide results, and this was not the case. In summary, whether the mean or highest PD-L1 core value was taken, and whether ICs or TCs were investigated, the number of positive tumors remained low.

### PD-L1 expression in BCG-naïve tumors does not predict outcome after BCG treatment

**Fig. 1:**
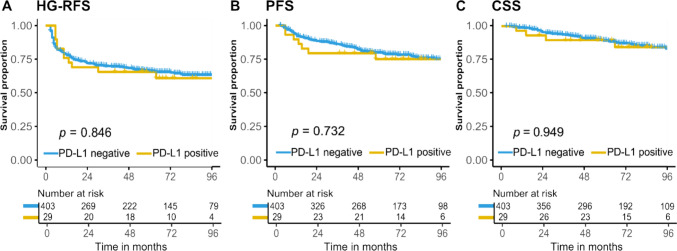
Kaplan–Meier estimates of **A** high-grade recurrence-free survival (HG-RFS), **B** progression-free survival (PFS), and **C** cancer-specific survival (CSS) as assessed by mean PD-L1 status of ≥ 5% in N = 432 BCG-naïve high-risk non-muscle invasive bladder cancer patients

We aimed to determine if PD-L1 expression was associated with a HG recurrence after adequate BCG treatment, which occured in 58/432 (13%) patients. Additionally, 79/432 (18%) had a recurrence of any grade/stage after BCG induction. PD-L1 expression was not associated with BCG treatment failure after adequate BCG (*p* = 0.782) nor with recurrence after BCG induction (*p* = 0.626). Importantly, investigating TCs or using different cut-offs (≥ 1% or ≥ 10% PD-L1 staining determines if a tumor is PD-L1 positive), or selecting only patients with a re-TURBT and detrusor muscle in the specimen, did not alter these results. Survival analyses showed that PD-L1 expression was not associated with 1-year HG-RFS (76% in PD-L1 positive vs. 79% PD-L1 negative tumors, *p* = 0.846). In addition, no differences were found for 2-year PFS (79% vs. 89%, *p* = 0.732) or 5-year CSS (90% vs. 91%, *p* = 0.949) (Fig. 1). Selecting the highest expressed PD-L1 core (≥ 5% ICs) did not alter these results (Figure S2). We conclude that PD-L1 protein expression is not associated with outcomes after BCG treatment in HR-NMIBC patients.

### PD-L1 expression in BCG-naïve HR-NMIBC is associated with aggressive clinical features

Previously studies have shown that PD-L1 is associated with BC stage progression [8–10]. In our study, we confirmed that 25 out of 29 PD-L1 positive tumors had invasive T1 disease as compared to four with Ta disease (Table S2, *p* = 0.015). Contrary to other series, we did not find PD-L1 staining in patients with solitary CIS [8]. Recently, we published on the importance of T1 substaging in HR-NMIBC, in which we showed that patients with extensive disease into the lamina propria had a higher risk of BCG treatment failure than patients with micro-invasive disease [15]. Yet no difference in PD-L1 expression was found between tumors with T1 micro- vs extensive invasion (*p* = 0.361). Nonetheless, positive PD-L1 expression was correlated with patients having received a re-TURBT (*p* = 0.003), larger tumors (*p* = 0.045), and the EAU highest-risk disease criteria (*p* = 0.010) (Table S2). In brief, aggressive clinical features were associated with higher PD-L1 expression.

### PD-L1 expression is more frequently observed in tumor recurrences

Next, we aimed to investigate PD-L1 expression in post-BCG tumor recurrences (N = 160). Overall results for ICs and TCs and different cut-offs, as well as for the mean expression of cores vs. the highest expressed core, are listed in Table S3. BCG treatment failures were highlighted (N = 128/160, 80%) as these tumors did not respond to BCG and might be candidates for anti-PD-(L)1 ICI therapy. PD-L1 was more frequently expressed post-BCG than in BCG-naive tumors (14% vs 7%, *p* = 0.012), regardless of whether a tumor was considered a BCG failure (14% vs 13%, *p* > 0.999). Finally, we investigated matching-pair tumors from patients with BCG-naïve tumors and tumor recurrences. In 19 out of 22 PD-L1 positive recurrences, matching BCG-naïve tissue was available, of which 18 were PD-L1 negative before BCG. In conclusion, high PD-L1 expression was more often observed in tumor recurrences as compared to BCG-naïve tumors.

## Discussion

We investigated if PD-L1 protein expression was associated with response to BCG in a large and unbiased cohort of HR-NMIBC patients (N = 432) who received adequate BCG therapy with long-term clinical follow-up. We found that PD-L1 expression was positive in only 7% of tumors, and this expression was not associated with outcomes after BCG treatment in BCG-naïve tumors. Neither a change in the recommended 5% cut-off in tumor-infiltrating ICs nor an investigation of TCs altered these results. In addition, we found that aggressive tumor features were associated with increased PD-L1 expression, consistent with studies showing that PD-L1 overexpression correlates with BC stage progression [8, 9].

Previous studies showed that PD-L1 expression did not correspond with response to BCG, but the study populations consisted of small sample sizes (N = 22 to 186), and in 4/6 studies, varying antibodies were used [16–21]. Of particular interest are two studies that provide dissimilar evidence. *Pierconti *et al., investigated SP142, SP263, and 22C3 PD-L1 mAbs in N = 65 primary CIS patients, of which N = 37 developed HG recurring disease [22]. The percentage of samples that were PD-L1 positive based on tumor and immune cells was similar for SP142 and 22C3. SP263 showed inconsistent staining patterns. Only 22C3 was associated with recurring disease, while SP263 and SP142 did not differ between responders and non-responders, identical to the results presented in this study. A study by *Kates *et al*.* in N = 63 BCG-naïve tumors showed that PD-L1 (both 22C3 and SP142), using the combined positive score, was overexpressed in BCG non-responders (N = 32) [23]. Because of the patchy distribution of PD-L1, a whole slide analysis was performed. The percentage of SP142 staining is up to 28%, which could be because investigators used a selected cohort overrepresenting non-responders and a different scoring system. Based on contradictory findings and our study which indicates that PD-L1 expression has no prognostic value, we consider PD-L1 unfit as a biomarker for response to BCG.

We found that patients who recurred more often showed high PD-L1 expression. In 22 BCG-treated NMIBC patients that were sampled pre- and post-BCG, enhanced PD-L1 expression in tumor recurrences was seen, suggesting a PD-L1-mediated resistance mechanism [24]. In contrast, *Boorjian *et al. showed that patients who received BCG had lower PD-L1 expression at the time of RC, while others found that SP142 was lower in patients with a refractory recurrence [16, 25]. A study of N = 761 urothelial carcinoma patients investigating clinicopathological correlations for SP142 found that no intravesical BCG treatment before PD-L1 testing was associated with positive PD-L1 expression [26]. These findings directly contradict our results, yet multiple caveats must be addressed for the latter studies. The first study included HR-NMIBC patients who had a mixture of primary and recurring disease and not all patients received BCG [25]. In the second study, the mean expression of PD-L1 was generally very low (1.5%), differences were minimal (3% vs 0.6%) and measured in only 14 patients [16]. The third study included nephroureterectomies and stage T2-T4 tumors, which biases the primary analysis, and there was no BCG information given [26]. These caveats make it difficult to draw definitive conclusions.

Beyond its role as a prognostic biomarker, PD-L1 has long been recognized as a therapeutic target. In Keynote-057 (Cohort A, CIS only, N = 101), PD-L1 status was high (38%) in BCG unresponsive patients [12]. Neither the success rate by PD-L1 status nor the specific IHC antibody was mentioned. Considering the low durable response at 3 months from pembrolizumab, the high costs of ICI, other successful and less costly treatments like gemcitabine/docetaxel instillations becoming available and our data showing limited PD-L1 expression (14–25%) in recurring tumors, we argue that PD-L1 monotherapy is not an attractive approach in patients that failed BCG treatment [27]. Preclinical work in syngeneic mice and rats inoculated with BC cell lines and treated with anti-PD-L1 ICI in conjunction with BCG led to a tumor weight reduction and increased cytotoxic T cell immune responses as compared to BCG alone [28, 29]. Studies investigating a combination of PD-1/PD-L1 and BCG seem a more logical way forward. Currently, several of these studies are ongoing (e.g. POTOMAC or CREST). A combination strategy might have the potential to change treatment paradigms if side effects are tolerated.

This study had several limitations. First, we only used the SP142 antibody, while SP142 has shown higher interobserver variability, lower concordance, and lower overall expression as compared to other companion diagnostics [21, 30–32]. SP142 should be primarily scored in tumor-infiltrating immune cells and not in tumor cells, which is done in other antibodies and may explain the disparate outcomes. Nonetheless, all studies concluded that SP142 is useful to evaluate PD-L1 status in BC. Plus, the use of a single antibody for all samples prevents antibody-related bias. We found a low prevalence of BCG-naïve cores staining positive for PD-L1, and a potential explanation is that TMAs do not capture the intricate heterogeneity of the tumor microenvironment (TME). Nonetheless, we performed a whole slide analysis with different cut-offs and assessment techniques, but the results remained unaltered. A third limitation was that multiple TMA cores were lost during the production of the TMA, which could have affected endpoint analyses. Albeit we used triplicate cores, we advise using > 1 mm core sizes in the future. Finally, we did not investigate PD-L1 in the context of the TME. Analysis of immune cells such as CD4 + , CD8 + , T regs, and (innate) immune cells might help explain (adaptive) immune resistance mechanisms. Although interesting, this study aimed to assess whether PD-L1 alone could be used as a biomarker for response to BCG treatment.

## Conclusions

IHC PD-L1 expression in BCG-naïve HR-NMIBC was not associated with BCG treatment failure. Based on low PD-L1 expression before BCG therapy, there seems to be no rationale for PD-L1 ICI monotherapy in BCG-naïve tumors. PD-L1 is higher in a subset of tumors that failed BCG treatment. More research is needed to determine the role of PD-L1 in tumors that failed BCG treatment, preferably within the context of the TME.

## Material and/or code availability

Only trivial statistical code was used for the analysis of the data.

## Supplementary Information

Below is the link to the electronic supplementary material.

Supplementary file1 (DOCX 764 KB)

## Data Availability

Clinical data cannot be uploaded due to privacy reasons.
