# Supplementary material for: PD-L1 expression in high-risk non-muscle invasive bladder cancer is not a biomarker of response to BCG
Source: World J Urol. 2025 Jan 3;43(1):57. doi: 10.1007/s00345-024-05392-5 (PMC11698758; doi:10.1007/s00345-024-05392-5)
Supplement: Supplementary file 1 — Supplementary file1 (DOCX 764 KB) [file 345_2024_5392_MOESM1_ESM.docx]

**Supplementary Material:**

*Supplementary methods (Patients)*

At data collection, we adhered to the EAU 2021 NMIBC guidelines. During clinical follow-up, urologists were recommended to adhere to the then applicable NMIBC guideline, which included follow-up withcystoscopy plus urine cytology every three months year 0–2, every six months year 2–5, and yearly afterward. Upper tract CT urography was performed at diagnosis and year 1. CT urography or ultrasound was performed every year thereafter. When intravesical recurrences were suspected, transurethral random biopsies were performed. (Lymph node) metastatic disease status was determined with a CT thorax/abdomen. Patients were classified as high-risk or as the highest subgroup according to the EAU guidelines NMIBC of 2017, as the 2021 guideline cannot be accurately applied when tumor size is missing. The highest-risk criteria included: multiple or large tumors (≥3cm), concomitant CIS, lymphovascular invasion (LVI), or specific variant histologies: micropapillary, sarcomatoid, nested, and neuroendocrine histology [1].

*Supplementary methods (TMA)*

Hematoxylin & eosin (HE) slides from primary tumors (*n*=509), re-TURBTs (*n*=266), recurrences (*n*=338), and benign biopsies (*n*=155) were centrally reviewed by the uropathologist as described previously [15]. Patients with low- or intermediate-risk disease or MIBC upon review, were excluded from analyses. HE review included selecting three regions of interest (ROI) for tissue microarray (TMA) construction. Preferred ROIs were invasive tumor areas with tumor-infiltrating lymphocytes (TILs). If absent, a high-grade (HG) tumor with tumor-infiltrating immune cells in close proximity to stroma was encircled. Formalin-fixed, paraffin-embedded (FFPE) tumor blocks from the entire cohort were obtained and HE slides were used as templates to construct TMAs in triplicate with a 1 mm diameter using the TMA Grand Master (3DHISTECH). For non-tumor normal samples from post-BCG biopsies, a single core was produced.

*Supplementary methods (Immunohistochemistry)*

IHC was performed on 4 µm whole slide sections from FFPE tissue blocks, on an accredited automated slide stainer (BenchMark ULTRA System, Ventana Medical Systems, Tucson, AZ, USA) according to the manufacturer’s instructions. Briefly, following deparaffinization and heat-induced antigen retrieval, the tissue samples were incubated with the monoclonal rabbit anti-PD-L1 antibody (VENTANA PD-L1 (SP142) Assay, Ventana Medical Systems, companion diagnostic for atezolizumab) for 32 min at 37 °C, followed by hematoxylin II counter stain for 12 min and a blue coloring reagent for 8 min. Visualization was obtained by the OptiView DAB IHC Detection Kit (Ventana Medical Systems). Each tissue slide contained FFPE tonsil as an on-slide positive control.

*Supplementary methods (IHC assessment)*

PD-L1/SP142 IHC was evaluated by two independent investigators, who were blinded for clinical outcomes during assessment. Cores were removed from analyses if these were deemed of insufficient quality. PD-L1 evaluation is defined as the proportion of tumor area occupied by PD-L1 expressing tumor-infiltrating immune cells (% IC) with punctate, linear, or circumferential membrane staining of any intensity or the percentage of PD-L1 expressing tumor cells (% TC) of any intensity. Staining was estimated in percentages; a consensus scoring was determined if percentages differed more than 5%. A ≥ 5% positivity cut-off was met according to the manufacturer’s instructions as mentioned in the package insert for the PD-L1/SP142 assay. A secondary analysis of ≥1% and ≥10% cut-off was also tested and we performed analyses with the highest expressing core only among the triplicate cores. The height of expression of the cores used in the analyses was calculated based on the mean percentages of both investigators. In cases in which multiple recurrences were available per patient, the highest PD-L1 expressing sample was selected for analysis. Because of known PD-L1 staining heterogeneity in previous studies, we also performed analyses with the highest expressing core only among the triplicate cores.

*Supplementary tables:*

**Table S1.** PD-L1 status in tumor-infiltrating immune cells and tumor cells in N=432 BCG-naïve high-risk non-muscle invasive bladder cancer. Results are shown for the mean expression of triplicate cores vs. the highest expressed core. In bold: the main cut-off (≥5%) as indicated by the SP142 PD-L1 protocol.

| **PD-L1 status** | ≥1% (mean cores) | ≥5% (mean cores) | ≥10% (mean cores) |
| --- | --- | --- | --- |
| ICs ^1^ | 87 (20%) | **29 (7%)** | 10 (2%) |
| TCs ^2^ | 41 (10%) | 19 (4%) | 9 (2%) |
| **PD-L1 status** | ≥1% (highest core) | ≥5% (highest core) | ≥10% (highest core) |
| ICs ^1^ | 181 (42%) | 69 (16%) | 40 (9%) |
| TCs ^2^ | 98 (23%) | 40 (9%) | 24 (6%) |

^1^ IC = tumor-infiltrating immune cells

^2^ TC = tumor cells.

**Table S2.** Clinicopathological frequencies and associations in low (N=403, <5%) versus high (N=29, ≥5%) PD-L1 expression in N=432 BCG-naïve high-risk non-muscle invasive bladder cancer patients.

| **Clinical characteristics** | **PD-L1 status** | | ***P* value** |
| --- | --- | --- | --- |
|  | **ICs <5% (N=403)** | **ICs ≥5% (N=29)** |  |
| Median age at diagnosis (IQR) | 70 (63-77) | 70 (63-77) | 0.835 |
| Female gender (%) | 87 (22) | 4 (14) | 0.479 |
| Active and former smoker (%) | 245 (61) | 15 (52) | 0.419 |
| Re-TURBT (%) | 237 (59) | 25 (86) | **0.003** |
| WHO stage (T1 vs Ta/Tis) (%) | 257 (64) | 25 (86) | **0.015** |
| WHO grade 3 (%) ^1^ | 349 (94) | 29 (100) | 0.390 |
| Carcinoma in situ (%) | 117 (29) | 6 (21) | 0.400 |
| Multifocal tumor (%) | 205 (51) | 16 (55) | 0.706 |
| Large tumor (%) ^1,2^ | 53 (44) | 8 (80) | **0.045** |
| Variant histology (%) ^1^ | 38 (15) | 6 (24) | 0.247 |
| Lymphovascular invasion (%) ^1^ | 13 (5) | 1 (4) | 1.000 |
| EAU high vs very high-risk (%) | 10 (2) | 19 (66) | **0.010** |
| BCG failure ^3^ (%) | 136 (34) | 11 (38) | 0.687 |
| Progression ^4^ (%) | 83 (21) | 7 (24) | 0.638 |
| Death from bladder cancer (%) | 52 (13) | 4 (14) | 0.780 |

^1^ Excluded patients with solitary carcinoma in situ. ^2^ Excluded patients with missing variables. ^3^ BCG failure as specified by EAU guidelines, which includes progression to MIBC, T1HG disease at 3 months and HG recurrences after adequate BCG or during BCG maintenance. ^4^ Progression includes patients with muscle-invasive, lymph node and distant metastatic disease. Abbreviations: BCG = Bacillus Calmette-Guérin; EAU = European Association of Urology; IQR = Interquartile Range; TURBT = Transurethral Resection of Bladder Tumor; WHO = World Health Organization.

**Table S3.** PD-L1 status in tumor-infiltrating immune cells and tumor cells in N=160 tumor recurrences. N=128/160 tumors were considered BCG failures according to the EAU guidelines. Results are shown for the mean expression of triplicate cores vs. the highest expressed core.

| **PD-L1 status** | **≥1% (mean cores)** | **≥5% (mean cores)** | **≥10% (mean cores)** |
| --- | --- | --- | --- |
| Tumor recurrence ^1^ |  |  |  |
| ICs | 45/160 (28%) | **22/160 (14%)** | 9/160 (6%) |
| TCs | 31/160 (19%) | 14/160 (9%) | 10/160 (6%) |
| BCG failure ^2^ |  |  |  |
| ICs | 37/128 (29%) | 17/128 (13%) | 7/128 (7%) |
| TCs | 22/128 (17%) | 10/128 (8%) | 6/128 (5%) |
| **PD-L1 status** | **≥1% (highest core)** | **≥5% (highest core)** | **≥10% (highest core)** |
| Tumor recurrence ^1^ |  |  |  |
| ICs | 79/160 (49%) | **40/160 (25%)** | 28/160 (18%) |
| TCs | 53/160 (33%) | 30/160 (19%) | 22/160 (14%) |
| BCG failure ^2^ |  |  |  |
| ICs | 66/128 (52%) | 33/128 (26%) | 22/128 (17%) |
| TCs | 43/128 (34%) | 21/128 (16%) | 15/128 (12%) |

^1^ Tumor recurrence of any stage or grade. ^2^ BCG failure as specified by major urology guidelines, which includes progression to MIBC, patients with T1HG disease at 3 months, and patients with HG recurrences after adequate BCG. In bold: the recommended cut-off of 5% ICs. Abbreviations: EAU = European Association of Urology; ICs = tumor-infiltrating immune cells; TCs = tumor cells.

*Supplementary figures:*

**
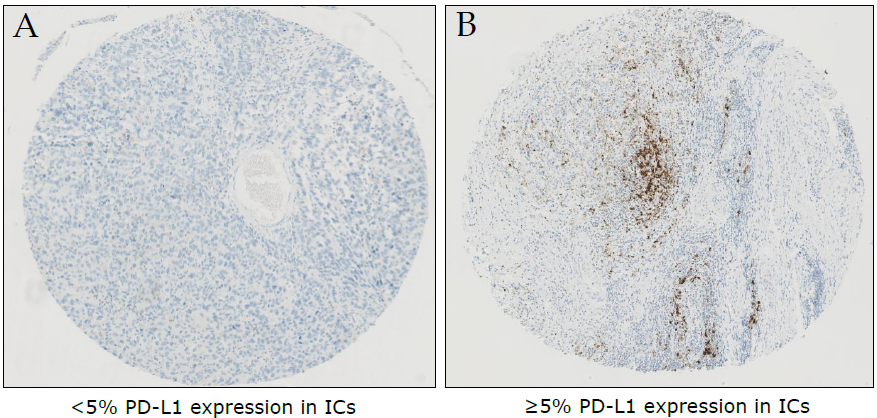
**

**Figure S1.** Example of PD-L1 (SP142) staining in TMAs. **(A)** Example of TMA core negative for PD-L1 staining. **(B)** Example of TMA core positive with high PD-L1 staining in ≥5% ICs. Abbreviations: ICs = tumor-infiltrating immune cells; TMA = Tissue Microarrays.

**
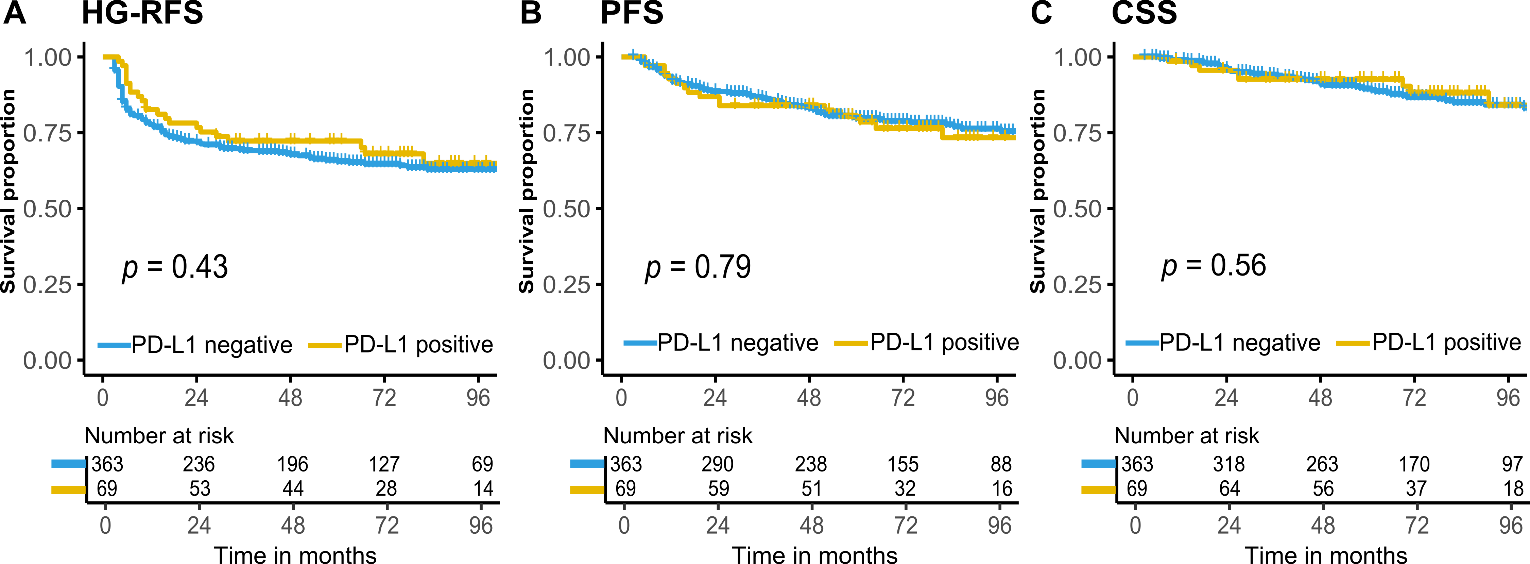
**

**Figure S2.** From left to right: Kaplan-Meier estimates of (A) high-grade recurrence-free, (B) progsion-free, and (C) cancer-specific survival as assessed by max PD-L1 ICs positivity status of ≥5% in N=432 BCG-naïve high-risk non-muscle invasive bladder cancer patients.
